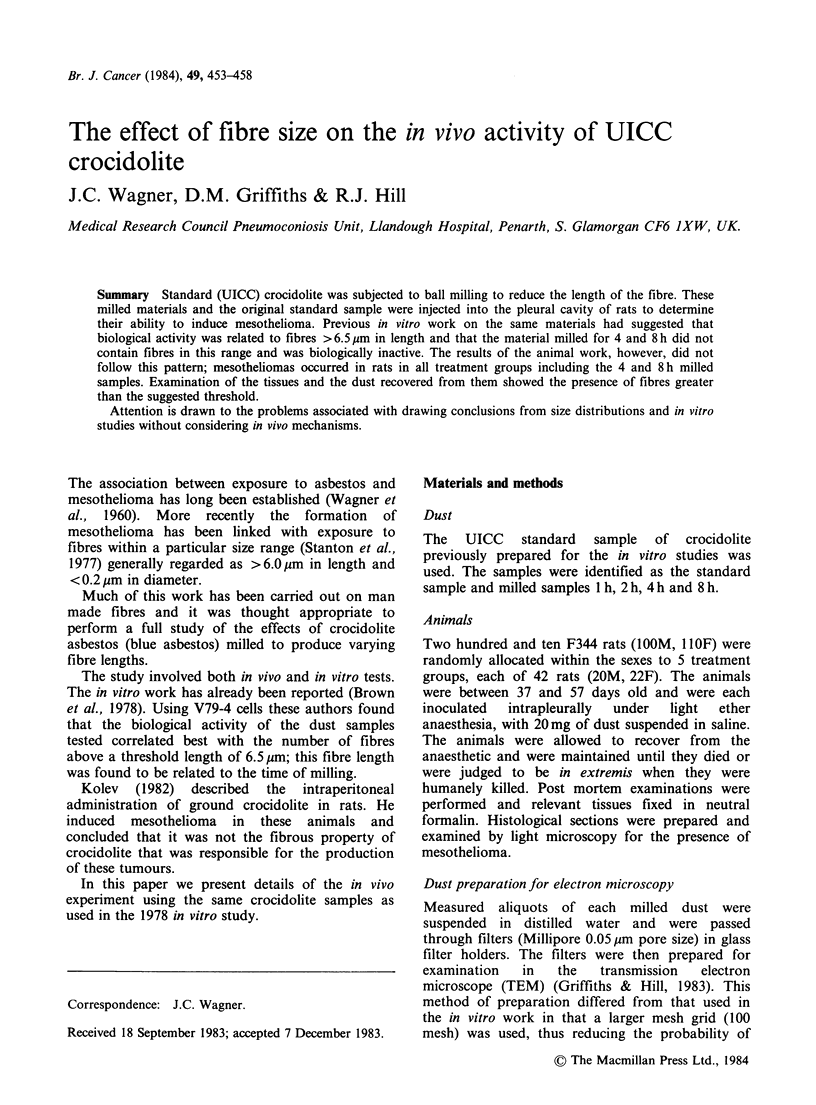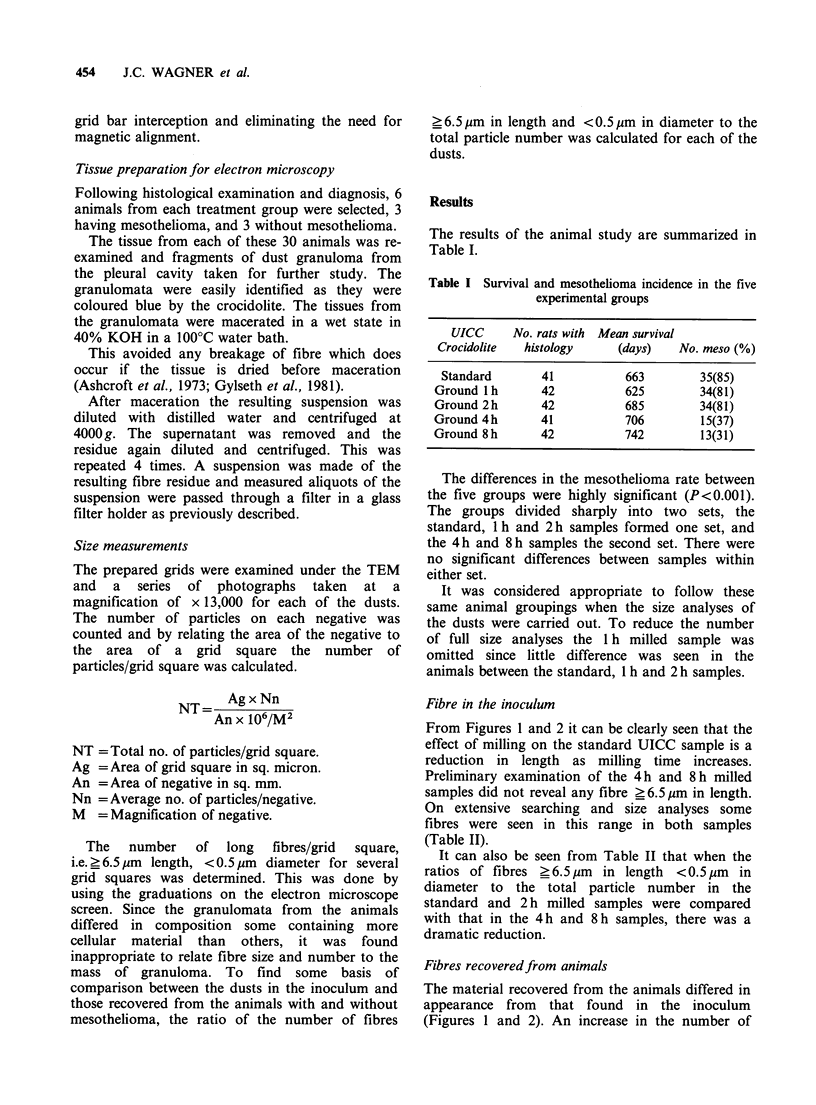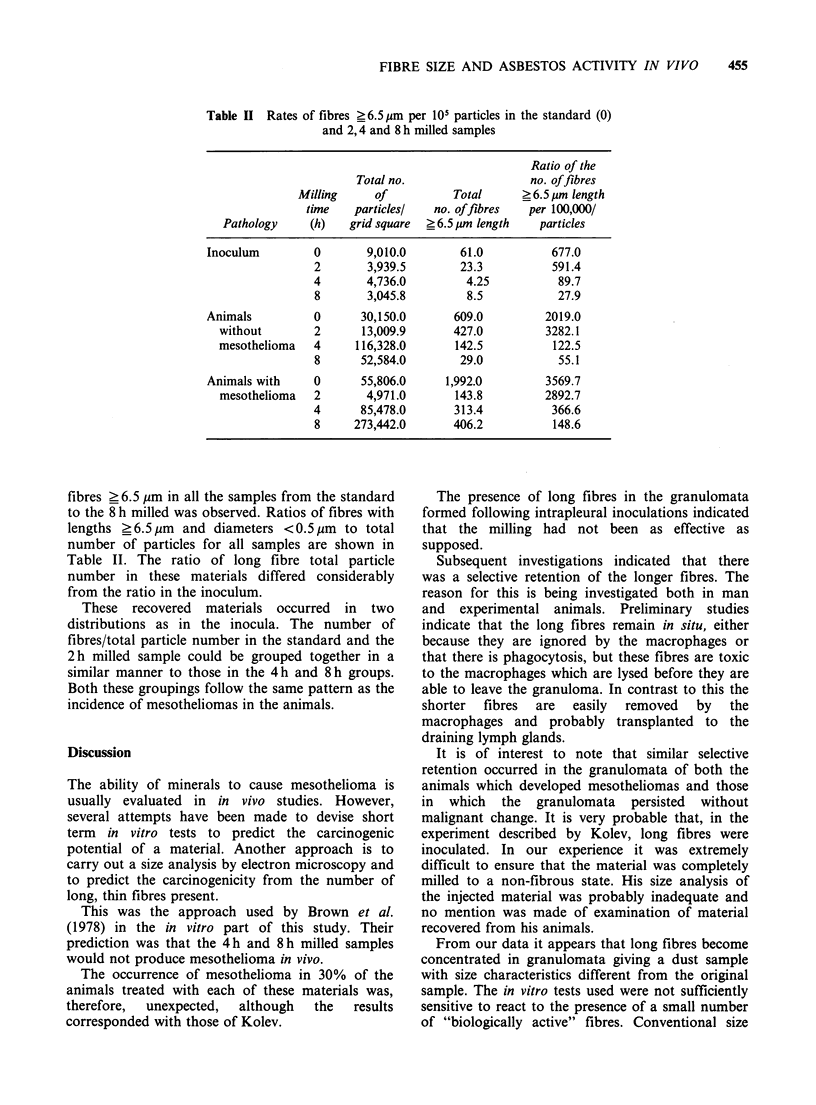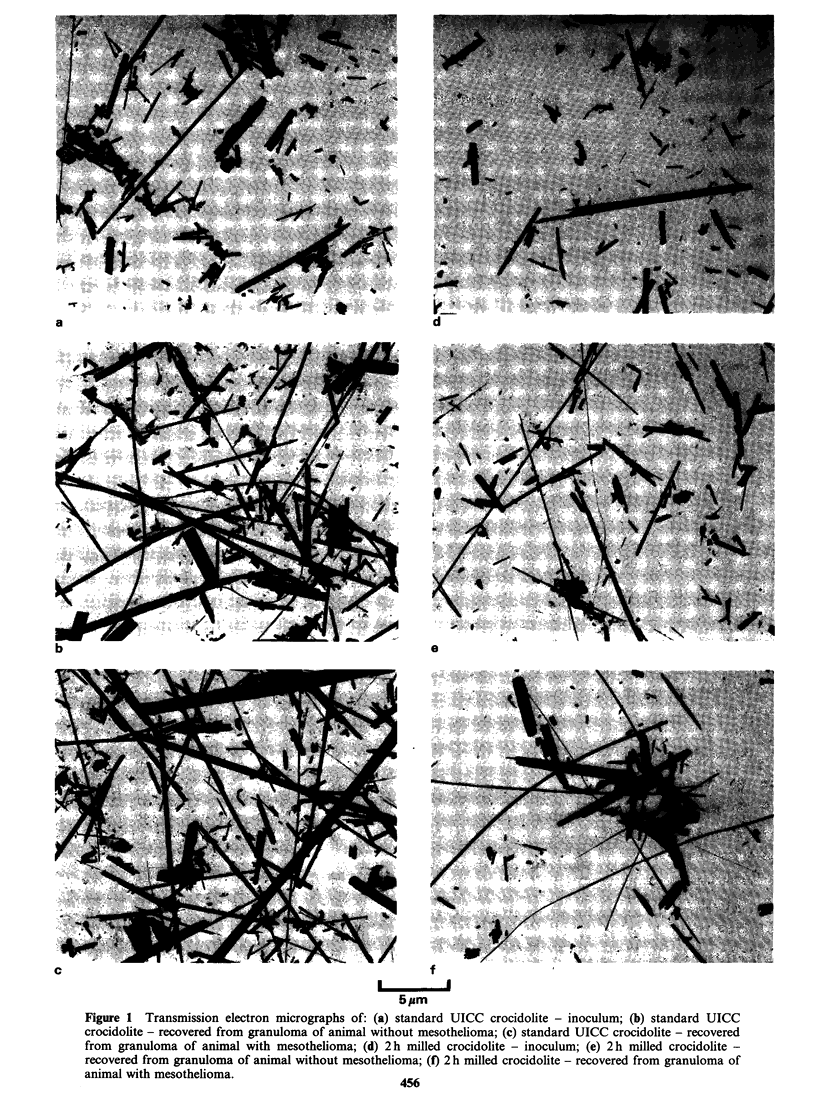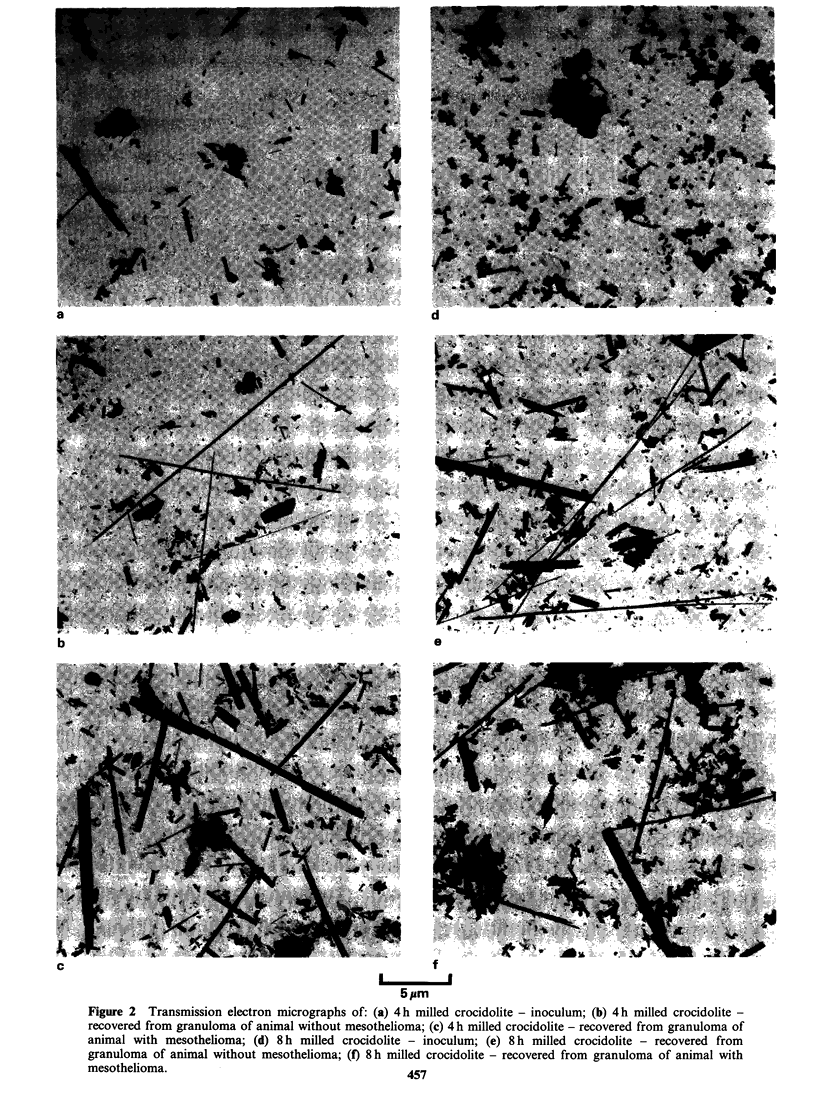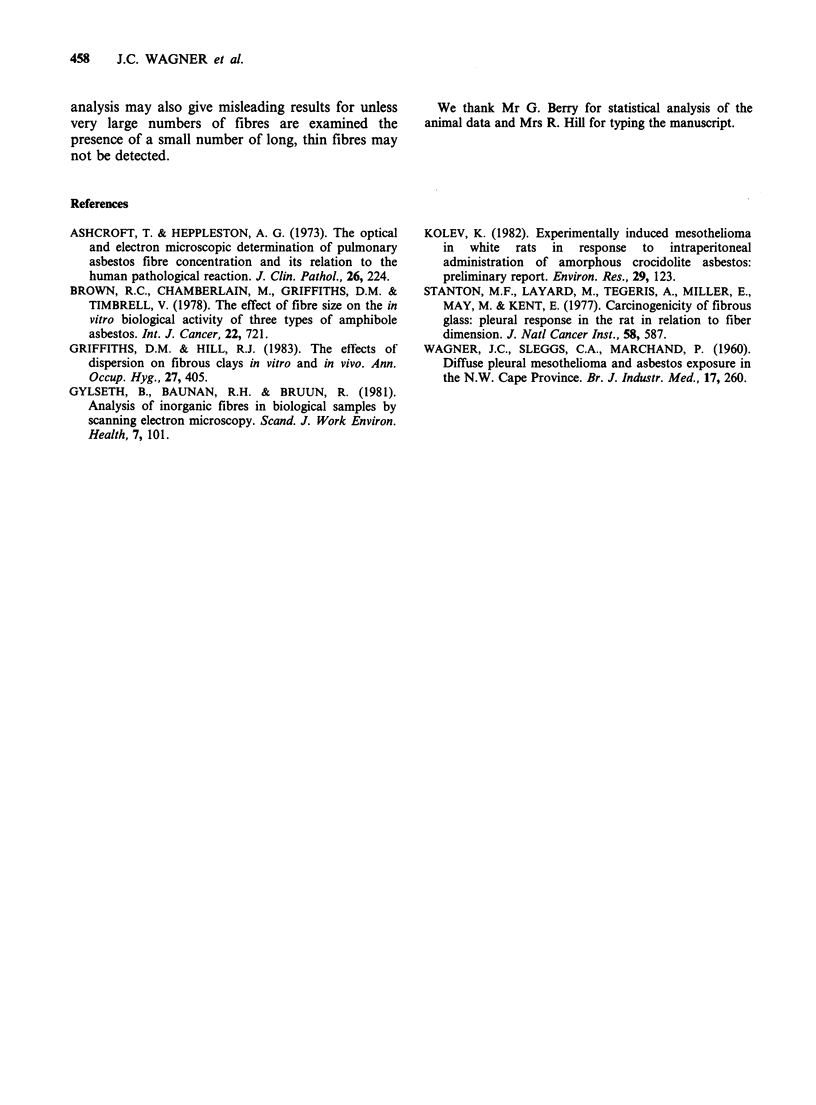# The effect of fibre size on the in vivo activity of UICC crocidolite.

**DOI:** 10.1038/bjc.1984.72

**Published:** 1984-04

**Authors:** J. C. Wagner, D. M. Griffiths, R. J. Hill

## Abstract

**Images:**


*Br. J. Cancer* (1984), **49**, 453–458

### The effect of fibre size on the *in vivo* activity of UICC crocidolite

J.C. Wagner, D.M. Griffiths & R.J. Hill

*Medical Research Council Pneumoconiosis Unit, Llandough Hospital, Penarth, S. Glamorgan CF6 1XW, UK.*

**Summary** Standard (UICC) crocidolite was subjected to ball milling to reduce the length of the fibre. These milled materials and the original standard sample were injected into the pleural cavity of rats to determine their ability to induce mesothelioma. Previous *in vitro* work on the same materials had suggested that biological activity was related to fibres $>6.5\,\mu m$ in length and that the material milled for 4 and 8 h did not contain fibres in this range and was biologically inactive. The results of the animal work, however, did not follow this pattern; mesotheliomas occurred in rats in all treatment groups including the 4 and 8 h milled samples. Examination of the tissues and the dust recovered from them showed the presence of fibres greater than the suggested threshold.

Attention is drawn to the problems associated with drawing conclusions from size distributions and *in vitro* studies without considering *in vivo* mechanisms.

The association between exposure to asbestos and mesothelioma has long been established (Wagner *et al.,* 1960). More recently the formation of mesothelioma has been linked with exposure to fibres within a particular size range (Stanton *et al.,* 1977) generally regarded as $>6.0\,\mu m$ in length and $<0.2\,\mu m$ in diameter.

Much of this work has been carried out on man made fibres and it was thought appropriate to perform a full study of the effects of crocidolite asbestos (blue asbestos) milled to produce varying fibre lengths.

The study involved both *in vivo* and *in vitro* tests. The *in vitro* work has already been reported (Brown *et al.,* 1978). Using V79-4 cells these authors found that the biological activity of the dust samples tested correlated best with the number of fibres above a threshold length of $6.5\,\mu m$; this fibre length was found to be related to the time of milling.

Kolev (1982) described the intraperitoneal administration of ground crocidolite in rats. He induced mesothelioma in these animals and concluded that it was not the fibrous property of crocidolite that was responsible for the production of these tumours.

In this paper we present details of the *in vivo* experiment using the same crocidolite samples as used in the 1978 *in vitro* study.

Correspondence: J.C. Wagner.

Received 18 September 1983; accepted 7 December 1983.

### Materials and methods

### *Dust*

The UICC standard sample of crocidolite previously prepared for the *in vitro* studies was used. The samples were identified as the standard sample and milled samples 1 h, 2 h, 4 h and 8 h.

### *Animals*

Two hundred and ten F344 rats (100M, 110F) were randomly allocated within the sexes to 5 treatment groups, each of 42 rats (20M, 22F). The animals were between 37 and 57 days old and were each inoculated intrapleurally under light ether anaesthesia, with 20 mg of dust suspended in saline. The animals were allowed to recover from the anaesthetic and were maintained until they died or were judged to be *in extremis* when they were humanely killed. Post mortem examinations were performed and relevant tissues fixed in neutral formalin. Histological sections were prepared and examined by light microscopy for the presence of mesothelioma.

### *Dust preparation for electron microscopy*

Measured aliquots of each milled dust were suspended in distilled water and were passed through filters (Millipore $0.05\,\mu m$ pore size) in glass filter holders. The filters were then prepared for examination in the transmission electron microscope (TEM) (Griffiths & Hill, 1983). This method of preparation differed from that used in the *in vitro* work in that a larger mesh grid (100 mesh) was used, thus reducing the probability of

© The Macmillan Press Ltd., 1984

grid bar interception and eliminating the need for magnetic alignment.

*Tissue preparation for electron microscopy*

Following histological examination and diagnosis, 6 animals from each treatment group were selected, 3 having mesothelioma, and 3 without mesothelioma.

The tissue from each of these 30 animals was re-examined and fragments of dust granuloma from the pleural cavity taken for further study. The granulomata were easily identified as they were coloured blue by the crocidolite. The tissues from the granulomata were macerated in a wet state in 40% KOH in a 100°C water bath.

This avoided any breakage of fibre which does occur if the tissue is dried before maceration (Ashcroft *et al.*, 1973; Gylseth *et al.*, 1981).

After maceration the resulting suspension was diluted with distilled water and centrifuged at 4000 *g*. The supernatant was removed and the residue again diluted and centrifuged. This was repeated 4 times. A suspension was made of the resulting fibre residue and measured aliquots of the suspension were passed through a filter in a glass filter holder as previously described.

*Size measurements*

The prepared grids were examined under the TEM and a series of photographs taken at a magnification of ×13,000 for each of the dusts. The number of particles on each negative was counted and by relating the area of the negative to the area of a grid square the number of particles/grid square was calculated.

$$NT = \frac{Ag \times Nn}{An \times 10^6/M^2}$$

NT = Total no. of particles/grid square.
Ag = Area of grid square in sq. micron.
An = Area of negative in sq. mm.
Nn = Average no. of particles/negative.
M = Magnification of negative.

The number of long fibres/grid square, i.e. $\geq 6.5\,\mu m$ length, $<0.5\,\mu m$ diameter for several grid squares was determined. This was done by using the graduations on the electron microscope screen. Since the granulomata from the animals differed in composition some containing more cellular material than others, it was found inappropriate to relate fibre size and number to the mass of granuloma. To find some basis of comparison between the dusts in the inoculum and those recovered from the animals with and without mesothelioma, the ratio of the number of fibres $\geq 6.5\,\mu m$ in length and $<0.5\,\mu m$ in diameter to the total particle number was calculated for each of the dusts.

### Results

The results of the animal study are summarized in Table I.

**Table I** Survival and mesothelioma incidence in the five experimental groups

| *UICC Crocidolite* | *No. rats with histology* | *Mean survival (days)* | *No. meso* (%) |
|---|---|---|---|
| Standard | 41 | 663 | 35(85) |
| Ground 1 h | 42 | 625 | 34(81) |
| Ground 2 h | 42 | 685 | 34(81) |
| Ground 4 h | 41 | 706 | 15(37) |
| Ground 8 h | 42 | 742 | 13(31) |

The differences in the mesothelioma rate between the five groups were highly significant ($P<0.001$). The groups divided sharply into two sets, the standard, 1 h and 2 h samples formed one set, and the 4 h and 8 h samples the second set. There were no significant differences between samples within either set.

It was considered appropriate to follow these same animal groupings when the size analyses of the dusts were carried out. To reduce the number of full size analyses the 1 h milled sample was omitted since little difference was seen in the animals between the standard, 1 h and 2 h samples.

*Fibre in the inoculum*

From Figures 1 and 2 it can be clearly seen that the effect of milling on the standard UICC sample is a reduction in length as milling time increases. Preliminary examination of the 4 h and 8 h milled samples did not reveal any fibre $\geq 6.5\,\mu m$ in length. On extensive searching and size analyses some fibres were seen in this range in both samples (Table II).

It can also be seen from Table II that when the ratios of fibres $\geq 6.5\,\mu m$ in length $<0.5\,\mu m$ in diameter to the total particle number in the standard and 2 h milled samples were compared with that in the 4 h and 8 h samples, there was a dramatic reduction.

*Fibres recovered from animals*

The material recovered from the animals differed in appearance from that found in the inoculum (Figures 1 and 2). An increase in the number of

**Table II** Rates of fibres $\geqq 6.5\,\mu m$ per $10^5$ particles in the standard (0) and 2, 4 and 8 h milled samples

| *Pathology* | *Milling time (h)* | *Total no. of particles/ grid square* | *Total no. of fibres $\geqq 6.5\,\mu m$ length* | *Ratio of the no. of fibres $\geqq 6.5\,\mu m$ length per 100,000/ particles* |
|---|---|---|---|---|
| Inoculum | 0 | 9,010.0 | 61.0 | 677.0 |
| | 2 | 3,939.5 | 23.3 | 591.4 |
| | 4 | 4,736.0 | 4.25 | 89.7 |
| | 8 | 3,045.8 | 8.5 | 27.9 |
| Animals without mesothelioma | 0 | 30,150.0 | 609.0 | 2019.0 |
| | 2 | 13,009.9 | 427.0 | 3282.1 |
| | 4 | 116,328.0 | 142.5 | 122.5 |
| | 8 | 52,584.0 | 29.0 | 55.1 |
| Animals with mesothelioma | 0 | 55,806.0 | 1,992.0 | 3569.7 |
| | 2 | 4,971.0 | 143.8 | 2892.7 |
| | 4 | 85,478.0 | 313.4 | 366.6 |
| | 8 | 273,442.0 | 406.2 | 148.6 |

fibres $\geqq 6.5\,\mu m$ in all the samples from the standard to the 8 h milled was observed. Ratios of fibres with lengths $\geqq 6.5\,\mu m$ and diameters $<0.5\,\mu m$ to total number of particles for all samples are shown in Table II. The ratio of long fibre total particle number in these materials differed considerably from the ratio in the inoculum.

These recovered materials occurred in two distributions as in the inocula. The number of fibres/total particle number in the standard and the 2 h milled sample could be grouped together in a similar manner to those in the 4 h and 8 h groups. Both these groupings follow the same pattern as the incidence of mesotheliomas in the animals.

### Discussion

The ability of minerals to cause mesothelioma is usually evaluated in *in vivo* studies. However, several attempts have been made to devise short term *in vitro* tests to predict the carcinogenic potential of a material. Another approach is to carry out a size analysis by electron microscopy and to predict the carcinogenicity from the number of long, thin fibres present.

This was the approach used by Brown *et al.* (1978) in the *in vitro* part of this study. Their prediction was that the 4 h and 8 h milled samples would not produce mesothelioma *in vivo*.

The occurrence of mesothelioma in 30% of the animals treated with each of these materials was, therefore, unexpected, although the results corresponded with those of Kolev.

The presence of long fibres in the granulomata formed following intrapleural inoculations indicated that the milling had not been as effective as supposed.

Subsequent investigations indicated that there was a selective retention of the longer fibres. The reason for this is being investigated both in man and experimental animals. Preliminary studies indicate that the long fibres remain *in situ*, either because they are ignored by the macrophages or that there is phagocytosis, but these fibres are toxic to the macrophages which are lysed before they are able to leave the granuloma. In contrast to this the shorter fibres are easily removed by the macrophages and probably transplanted to the draining lymph glands.

It is of interest to note that similar selective retention occurred in the granulomata of both the animals which developed mesotheliomas and those in which the granulomata persisted without malignant change. It is very probable that, in the experiment described by Kolev, long fibres were inoculated. In our experience it was extremely difficult to ensure that the material was completely milled to a non-fibrous state. His size analysis of the injected material was probably inadequate and no mention was made of examination of material recovered from his animals.

From our data it appears that long fibres become concentrated in granulomata giving a dust sample with size characteristics different from the original sample. The *in vitro* tests used were not sufficiently sensitive to react to the presence of a small number of "biologically active" fibres. Conventional size

**Figure 1** Transmission electron micrographs of: (**a**) standard UICC crocidolite – inoculum; (**b**) standard UICC crocidolite – recovered from granuloma of animal without mesothelioma; (**c**) standard UICC crocidolite – recovered from granuloma of animal with mesothelioma; (**d**) 2 h milled crocidolite – inoculum; (**e**) 2 h milled crocidolite – recovered from granuloma of animal without mesothelioma; (**f**) 2 h milled crocidolite – recovered from granuloma of animal with mesothelioma.

**Figure 2** Transmission electron micrographs of: (**a**) 4 h milled crocidolite – inoculum; (**b**) 4 h milled crocidolite – recovered from granuloma of animal without mesothelioma; (**c**) 4 h milled crocidolite – recovered from granuloma of animal with mesothelioma; (**d**) 8 h milled crocidolite – inoculum; (**e**) 8 h milled crocidolite – recovered from granuloma of animal without mesothelioma; (**f**) 8 h milled crocidolite – recovered from granuloma of animal with mesothelioma.

analysis may also give misleading results for unless very large numbers of fibres are examined the presence of a small number of long, thin fibres may not be detected.

We thank Mr G. Berry for statistical analysis of the animal data and Mrs R. Hill for typing the manuscript.

### References

ASHCROFT, T. & HEPPLESTON, A. G. (1973). The optical and electron microscopic determination of pulmonary asbestos fibre concentration and its relation to the human pathological reaction. *J. Clin. Pathol.*, **26**, 224.

BROWN, R.C., CHAMBERLAIN, M., GRIFFITHS, D.M. & TIMBRELL, V. (1978). The effect of fibre size on the *in vitro* biological activity of three types of amphibole asbestos. *Int. J. Cancer*, **22**, 721.

GRIFFITHS, D.M. & HILL, R.J. (1983). The effects of dispersion on fibrous clays *in vitro* and *in vivo*. *Ann. Occup. Hyg.*, **27**, 405.

GYLSETH, B., BAUNAN, R.H. & BRUUN, R. (1981). Analysis of inorganic fibres in biological samples by scanning electron microscopy. *Scand. J. Work Environ. Health*, **7**, 101.

KOLEV, K. (1982). Experimentally induced mesothelioma in white rats in response to intraperitoneal administration of amorphous crocidolite asbestos: preliminary report. *Environ. Res.*, **29**, 123.

STANTON, M.F., LAYARD, M., TEGERIS, A., MILLER, E., MAY, M. & KENT, E. (1977). Carcinogenicity of fibrous glass: pleural response in the rat in relation to fiber dimension. *J. Natl Cancer Inst.*, **58**, 587.

WAGNER, J.C., SLEGGS, C.A., MARCHAND, P. (1960). Diffuse pleural mesothelioma and asbestos exposure in the N.W. Cape Province. *Br. J. Industr. Med.*, **17**, 260.